# Comparison of dietary acid load score between celiac patients and healthy population

**DOI:** 10.1186/s40795-022-00512-z

**Published:** 2022-03-01

**Authors:** Zeinab Nikniaz, Reza Mahdavi, Mojgan Akhavan Sabbagh, Leila Nikniaz, Masood Shirmohammadi

**Affiliations:** 1 Liver and Gastrointestinal Diseases Research Center, Tabriz University of Medical Sciences, Tabriz, Iran; 2 Nutrition Research Center, Tabriz University of Medical Sciences, Tabriz, Iran; 3 Student Research Committee, Tabriz University of Medical Sciences, Tabriz, Iran; 4 Tabriz Health Services Management Research Center, Tabriz University of Medical Sciences, Tabriz, Iran

**Keywords:** Celiac disease, Dietary acid load, Gluten-free diet, NEAP, PRAL

## Abstract

**Background and aims:**

In the present study, we assessed the dietary acid load in adult celiac patients and compared it with that of the healthy population to provide more specific dietary recommendations for celiac patients.

**Methods:**

This study was a cross-sectional study that included 130 celiac patients and 462 Non-celiac participants. The 80-item semi-quantitative food frequency questionnaire was used to obtain dietary data. Based on the dietary data, the dietary acid load (DAL), Potential renal net acid load (PRAL), and net endogenous acid production (NEAP) were calculated.

**Results:**

The mean PRAL value is negative in the celiac group but positive in the general population. There was a significant difference in the PRAL score between celiac patients and the general population (*p* < 0.001). The mean NEAP and DAL score were significantly lower in the celiac group compared with the healthy population (*P* < 0.001). There were no significant differences between gluten-free diet adherents and non-adherents regarding the PRAL, NEAP, and DAL values (*P* > 0.05).

**Conclusion:**

We showed that the patients with celiac disease had a significantly less acidogenic diet compared with that of the general population. So, following dies low in gluten may be associated with less acid production spacially in populations at risk of acid/base imbalance.

## Introduction

Celiac disease (CD) is an autoimmune disease of the small intestine that presents in genetically susceptible individuals by consuming prolamins. Prolamins are found in wheat, barley, and rye [1]. The disease symptoms are not restricted to the gastrointestinal tract and different extra-intestinal manifestations including anemia, osteopenia, and weight loss occurred [1]. Moreover, these patients are at increased risk of developing type-1-diabetes [2], fractures [3], and cancers [4].

Lifelong adherence to the gluten-free diet (GFD) is the only available treatment for celiac patients [1]. In this diet, all sources of gluten are omitted from the diet, and alternative gluten-free products are added. Considering the different compositions of gluten-free alternatives and also changes in the entire diet of celiac patients, the diet quality of celiac patients is of concern. In this regard, some studies have assessed the diet quality of celiac patients using different indices. Morreale et al. showed that the mean score of the Mediterranean diet was significantly lower in celiac patients [5]. In our previous studies, we showed that in comparison with the healthy population, patients with celiac disease had significantly higher healthy eating index scores [6] and lower diet diversity scores [7].

The dietary acid load (DAL) is another index that is frequently used for the evaluation of diet quality in different populations. In various investigations, DAL has been estimated based on dietary data and calculating the Potential renal net acid load (PRAL) [8] and net endogenous acid production (NEAP) [9]. In different studies, the negative effect of high dietary acid load on cardiometabolic risk factors [10–12], serum fasting glucose [10], bone mineral status [13] have been shown.

Considering that celiac patients are at increased risk of these health-related conditions, it is important to assess the dietary acid load of a gluten-free diet and compare it with that of a healthy population. This could provide pivotal information for physicians and nutritionists to deliver more specific recommendations for celiac patients to increase their diet quality, health-related quality of life, and prevent celiac complications. Thus, for the first time, we aimed to assess the dietary acid load in adult celiac patients and compare it with that of the healthy population.

## Materials and methods

This investigation was a cross-sectional study in which the celiac patients were randomly selected from the East-Azerbaijan, Iran CD registry database. The inclusion criteria were as follows: age 20–55 years old, diagnosis of celiac according to biopsy report, and following GFD for at least one year. All patients are registered in the CD database. The patients who could not communicate with the interviewer or had other concomitant diseases were excluded.

As a general population, we used the data collected in the lifestyle promotion project (LPP) conducted in East Azerbaijan-Iran for the evaluation of lifestyle risk factors. We described the detailed method of participants' recruitment in our previous publication [14]. For this study, the data of 462 healthy participants with the age of 20–55 years old were included in the statistical analysis. The participants with known diabetes mellitus, CD, or other diseases that affect their diet were excluded from the analysis.

## Data collection

The author-designed checklist was used for obtaining demographic characteristics. The same instruments were used for measuring weight (Seca weighing scale) and height (stadiometer fixed to the wall) in both celiac patients and the general population. Body mass index (BMI) was calculated by dividing weight (kg) by height (m^2^). A BMI of less than 18.5 was considered as underweight, 18.5–24.99 was normal weight, and ≥ 25 was overweight.

An expert dietitian has obtained the dietary intake of protein, Potassium, Magnesium, Phosphorus, and Calcium using a Semi-quantitative food frequency questionnaire (FFQ). The questionnaire was validated previously in the East-Azerbaijan population [15]. For assessing the dietary intake of celiac patients, gluten-free items were also added to FFQ. The Iranian modified Nutritionist IV software was used for the determination of protein and micronutrient content.

## Dietary acid Load scores estimation

Three scores of dietary acid load including Net endogenous acid production (NEAP), Potential renal acid load (PRAL), and dietary acid load (DAL) were derived from estimations of several nutrient intakes [17].

1. PRAL (mEq/day) = 0.49 × protein (g) + 0.037 × phosphorus (mg) – 0.021 × potassium (mg) – 0.026 × magnesium (mg) – 0.013 × calcium (mg)
2. NEAP (mEq/day) =  − 10.2 + 54.5 (protein intake [g/d] ÷ potassium intake [mEq/d])
3. DAL (mEq/day) = PRAL + (body surface area [m2] × 41 [mEq/day]/1.73 m2)

Body surface area was calculated using the following formula: 0.007184 × height 0.725 × weight 0.425

## Assessing adherence to the CD

Adherence to the GFD for CD participants was determined by the Persian version of the celiac disease adherence test. This questionnaire was previously validated in our population. Patients with a score of less than 13 were considered good adherents [16].

## Statistical analysis

For statistical analyses, SPSS V 22 was used. Kolmogorov–Smirnov was used to verify the normality assumption. The independent t-test, chi-square, and Fisher exact tests were used for comparison of the general and anthropometric characteristics between groups. The one-way ANCOVA was used for comparing the dietary acid load scores between groups by adjusting to confounding factors such as age, sex, BMI, and energy intake. A significance level of 0.05 was used.

## Results

**Fig. 1:**
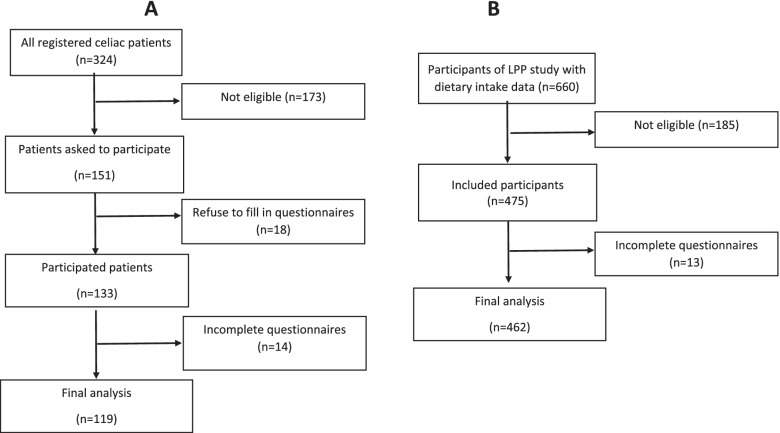
study enrolment flow chart Aceliac patients enrolment Bhealthy population enrolment

In the present study, the data of 14 patients with celiac disease were not included in the final analysis because of incomplete questionnaires (Fig. 1). The demographic and clinical information of participants stratified by the group is presented in Table 1. There were no significant differences between groups regarding age (*p* = 0.07) and sex distribution (*p* = 0.45). However, some anthropometric characteristics including weight and BMI were significantly lower in celiac patients compared with that of the general population (*P* < 0.05).

**Table 1:** The demographic and clinical characteristics of

Variables	Celiac patients (*n* = 119)	Healthy population (*n* = 462)	*p*-value
Age (years)	36.70 ± 9.46	39.75 ± 11.32	0.07
Sex (M:F)	22.7/77.3	44.9/54.9	0.45
Weight (kg)	63.16 ± 11.90	73.56 ± 13.01	< 0.001
Height (cm)	163.01 ± 10.13	163.89 ± 9.95	0.38
BMI (kg/m^2^)	23.76 ± 3.73	27.35 ± 4.76	< 0.001
Underweight	7.6	1.9	0.04
Normal weight	57.1	31.3	< 0.001
Overweight/obese	35.3	66.8	< 0.001
Disease duration (years)	6.41 ± 8.17	-	-

*BMI*: Body mass index

*P*-value of independent t-test

The PRAL, NEAP, and DAL values are reported in Table 2. As can be seen, the mean PRAL value was negative in the celiac group and it was positive in the general population. There was a significant difference in the PRAL score between celiac patients and the general population (*p* < 0.001).

**Table 2:** Comparison of dietary acid load between celiac patients and healthy population

Variables	Celiac patients			Healthy population	*p*-value*	*p*-value**
	**Total**	**adherents**	**Non-adherents**			
**PRAL score**					0.15	< 0.001
** Mean**	-36.30	-24.21	-37.55	36.91		
** SD**	31.34	20.34	34.94	55.59		
** Median**	-30.18	-21.15	-29.57	32.25		
** Min–Max**	-204.05, 3.96	-68.77, -1.37	-204.05, 3.96	-326.15, 371.29		
**NEAP score**					0.55	< 0.001
** Mean**	23.91	27.59	23.21	63.08		
** SD**	10.50	8.66	11.25	20.95		
** Median**	23.23	30.97	23.33	63.27		
** Min–Max**	0.03, 59.78	14.79, 42.44	0.03, 59.78	-3.96, 137.34		
** DAL score**					0.11	< 0.001
** Mean**	18.68	28.49	17.79	57.01		
** SD**	18.70	11.77	20.74	27.22		
** Median**	21.78	31.88	21.78	57.19		
** Min–Max**	-77.55, 47.37	55.53, 40.16	-77.55, 47.37	-147.97, 255.43		

*DAL* dietary acid load; *PRAL* Potential renal net acid load; *NEAP* net endogenous acid production

^*^P-value of ANCOVA comparing adherent and non-adherent celiac patients adjusted for age, sex, BMI, energy intake; disease duration, and treatment duration

^**^P-value of ANCOVA comparing celiac disease and healthy population adjusted for age, sex, BMI, and energy intake

The mean NEAP and DAL score were significantly lower in the celiac group compared with the healthy population (*P* < 0.001).

According to ANCOVA analysis, after adjusting for age, sex, BMI, energy intake, disease duration, and treatment duration, there were no significant differences between gluten-free diet adherents and non-adherents regarding the PRAL, NEAP, and DAL values (*P* > 0.05).

## Discussion

Constringing that the gluten-free diet should be followed strictly lifelong, and owing to this fact that the type of diet can affect the body by providing acid or base precursors [17], it is important to assess diet quality in celiac patients and compare it with that of the general population to provide the specific recommendation for this group. In this regard in the present study, we showed that the patients with celiac disease had significantly less acidogenic diet compared with that of the general population. This finding may be partly due to the gluten-free content of celiac patients. Interventional studies showed that a diet high in wheat gluten is associated with higher acid production [18, 19]. Another explanation for this observation may be due to the high consumption of fruits, vegetables, and dairies, and low consumption of protein and grains in Iranian patients with celiac disease [20]. In our previous study on the same population, we showed that patients with celiac disease had more consumption of fruits, vegetables, and dairy products [6]. It has been indicated that a high lacto-vegetarian diet was associated with reduced net acid excretion. Although, it has been indicated that animal proteins had significantly more acidogenic properties due to their phosphorus content, dairy products because of their calcium contents, had a more alkalotic effect [21]. In addition, celiac patients had a significantly lower amount of seafood and plant protein consumption. Studies showed that protein consumption irrespective of its source (plant or animal) had a significant role in increasing acid production [22]. Moreover, due to dietary restrictions of a gluten-free diet, the patients with celiac disease had a significantly lower amount of cereal consumption. On the other hand, while patients with celiac disease had no restriction on the consumption of meat products, due to the high price of these products in Iran, their consumption was very low [7], especially among population such as celiac patients that should spend more money on gluten-free products [23]. Rodrigues Neto Angeloco et al. showed that a diet with a decreased protein content and increased consumption of fruits and vegetables is associated with a lower dietary acid load [17].

It has been indicated that the acidogenic diet was associated with insulin resistance, diabetes, hypertension, chronic kidney disease, bone disorders, and low muscle mass [21]. Considering that the patients with celiac disease were at risk of developing these diseases, following a gluten-free diet with its alkalotic properties could prevent complications in celiac patients.

The present study had some limitations. For obtaining the dietary consumption of Potassium, Phosphorus, Magnesium, Calcium, and protein, we used FFQ. The limitations of this questionnaire such as recall bias may have affected some of the results. However, we used the validated FFQ which was also modified to use in celiac patients. Further, instead of recruiting a specific control group, we used the data of the previous study conducted on the general population of East-Azerbaijan, so we are not confident that all participants in the healthy population group, are free of disease. Moreover, both celiac patients and the general population are from East-Azerbaijan, Iran. So, their dietary habits may differ from those of other populations. This limits the generalizability of our findings.

## Conclusion

According to the results, we showed that the patients with celiac disease in East-Azerbaijan, Iran had a significantly less acidogenic diet compared with that of the general population So, following dies low in gluten may be associated with less acid production spacially in populations at risk of acid/base imbalance. However, for a precise conclusion, future studies should apply a more valid instrument for obtaining dietary intake data.

## Data Availability

Data will be available upon reasonable request from corresponding author Zeinab Nikniaz.
